# Effect of Hydrofluoric Acid Concentration on Bond Strength to Glass-Ceramics: A Systematic Review and Meta-Analysis of In-Vitro Studies

**DOI:** 10.3290/j.jad.b4646943

**Published:** 2023-11-17

**Authors:** Vitaliano Gomes de Araújo-Neto, Caio Felipe de Almeida Nobre, Mariana Itaborai Moreira Freitas, Renally Bezerra Wanderley Lima, Mario Alexandre Coelho Sinhoreti, Altair Antoninha Del Bel Cury, Marcelo Giannini

**Affiliations:** a PhD Student, Department of Restorative Dentistry, Piracicaba Dental School, University of Campinas. Piracicaba, Brazil. Conceptualization, performed database search, wrote the manuscript.; b PhD Student, Department of Restorative Dentistry, Piracicaba Dental School, University of Campinas. Piracicaba, Brazil. Conceptualization, performed database search, wrote the manuscript.; c Department of Prosthodontics and Periodontology, Piracicaba Dental School, University of Campinas. Piracicaba, Brazil.; d Professor, Department of Restorative Dentistry, Federal University of Paraíba. João Pessoa, PB. Brazil. Conceptualization, performed database search, statistical analysis, software, wrote the manuscript.; e Professor, Department of Restorative Dentistry, Piracicaba Dental School, University of Campinas. Piracicaba, Brazil. Conceptualization, statistical analysis, wrote, reviewed and edited the manuscript.; f Professor, Department of Prosthodontics and Periodontology, Piracicaba Dental School, University of Campinas. Piracicaba, Brazil.

**Keywords:** ceramics, dental bonding, shear strength

## Abstract

**Purpose:**

To conduct a systematic review and meta-analysis of in-vitro bond strength to glass-ceramics using hydrofluoric acid (HF) at lower (<5%) and higher (>5%) concentrations ([HF]) to treat ceramic surfaces.

**Methods:**

Systematic searches were carried out in PubMed, Scopus, LILACS, and Web of Science for articles published through July 2021, and a meta-analysis was performed to estimate the combined effect by comparing the differences between the standardized means of the bond strengths of the evaluated materials.

**Results:**

In total, 943 articles were found, of which 17 studies were selected for qualitative analysis and 12 for quantitative analysis. The bond strength to glass-ceramics using 4% to 5% HF did not differ from that using 7% to 10% HF for the following HF etching times and glass-ceramic materials: 20 s for lithium-disilicate (Z = 0.65, p = 0.51), 60 s for feldspathic (Z = 0.53, p = 0.60), and 60 s for leucite (Z = 0.72, p = 0.35).

**Conclusion:**

The lower concentration HF (<5%) etchant is a reliable surface treatment for adhesive bonding to glass-ceramics with satisfactory bond strength in short-term evaluations.

Dental ceramics are used in oral rehabilitation because they provide satisfactory esthetics, biocompatibility, and mechanical properties.^5,38,67^ The many types of dental ceramics are classified by material composition. For instance, feldspathic ceramics, leucite-reinforced ceramics, lithium-disilicate glass-ceramics, zirconia-reinforced lithium-silicate glass-ceramics, and polymer-infiltrated hybrids incorporate a glass phase that mimics the appearance of natural human teeth, which is desirable for indirect restorations.^13,21,25,67^

Reliable adhesive bonding to glass-ceramics is a crucial factor in the long-term success of indirect restorations.^18,22^ Several surface treatments for bonding glass-ceramics are presented in the literature,^19^ including chemical etching with hydrofluoric acid (HF),^59^ physical/chemical methods (alumina air abrasion, silicatization),^2^ and silane application.^32^ Sandblasting with 30- to 50-μm alumina abrasive particles can increase the surface roughness of ceramics^27,40^ but is not indicated for glass-ceramics because it reduces its flexural strength and leads to premature failures.^1^ Etching with hydrofluoric acid selectively attacks and removes parts of the glassy structure^3^ to increase free surface energy and improve the bond strength of composite cement with etched and silanated glass-ceramics. Thus, hydrofluoric acid etching is used to produce microretentions on glass-ceramic surfaces, and variations in concentrations (HF) and etching times have been used to produce different surface irregularity patterns.^9,13,62^ However, with higher concentrations of HF (>5%) and extended application times (20 to 160 s), the glass-ceramic matrix dissolves,^46^ thus compromising the mechanical properties and clinical longevity of the restoration, especially when using thin ceramic veneers.^13,35,68^

Previous laboratory studies have evaluated whether lower HF (<5%) can substitute higher HF (>5%) etchants, but these results are controversial and inconclusive.^9,24,46^ Therefore, this study aimed to conduct a systematic review and meta-analysis of the in-vitro bond strength of composite cement to glass-ceramics when using lower HF (<5%) etchants, compared with higher HF (>5%) etchants.

## Methods

This systematic review was reported following the guidelines of the PRISMA 2020 statement (Preferred Reporting Items for Systematic Reviews and Meta-Analyses)^40^ and was registered in the OFS database under the DOI number 10.17605/OSF.IO/WQDU8, available at https://osf.io/qg49x.

The research question set for the development of this study was: Are lower-concentration HF (<5%) etchants a suitable alternative to higher-concentration HF (>5%) etchants for glass-ceramic etching?

### Eligibility Criteria

The inclusion criteria for articles were: in-vitro studies using lower-concentration HF (<5%) etchants for glass-ceramic surface treatment (compared with higher-concentration HF, >5%); studies using primers specific to glass-ceramics; and studies reporting bond strength means and standard deviations (expressed in MPa) obtained by shear, microshear, tensile, or microtensile tests. In studies where information was missing, the authors were contacted and included in the study if the unpublished data was provided.

The exclusion criteria for articles were: studies using only silane-containing universal adhesives as pre-treatment for glass-ceramics; studies using exclusively hydrofluoric acid, experimental materials, aluminum oxide particles, plasma, or laser as surface treatment; and publications in the form of editor’s letters, comprehensive reviews, case reports, case series, editorials, consensus papers, and congress abstracts.

### Information Sources and Search Strategy

Four electronic databases (PubMed, Scopus, LILACS, and Web of Science) were systematically searched from April 1989 to July 2021 without language or time restrictions. To systematize the search, three of the authors were previously standardized. Medical subject heading (MeSH) terms, text words, MeSH synonyms, related terms, and free terms (see Table 1) were included. The terms were combined with the Boolean operators ‘AND’ and ‘OR’ while respecting database syntax rules (see supplementary material). Additional articles were collected manually from references of articles found through the search.

**Table 1:** Methodological data from included studies

Author	Year	Country	Number of samples	Ceramic	Surface treatment HF	Silane	Etching time (s)	Composite cement/Resin composite	Methodology	Aging/storage
Saraçoğlu [52]	2004	Turkey	10	Alumina-reinforced ceramic (IPS Empress, Ivoclar; Schaan, Liechtenstein)	4.9 and 9.5	Ultradent (South Jordan, UT, USA)	10, 20, and 40	Opal Luting Composite (3M Oral Care; St Paul, MN, USA)	Shear bond strength (MPa)	24 h
Venturini [61]	2015	Brazil	5	Feldspathic ceramic (VITA Mark II; Vita Zahnfabrik, Bad Säckingen, Germany)	1, 3, 5, and 10	ESPE-Sil (3M Oral Care; Seefeld, Germany)	60	RelyX ARC (3M Oral Care)	Microtensile bond strength (μTBS)	12,000 thermocycles
Sundfeld [58]	2015	Brazil	6	Lithium-disilicate (IPS e.max Press Ivoclar) and leucite-based glass ceramic (IPS Empress Esthetic, Ivoclar)	1, 2.5, 5, 7.5, 10, and 15	RelyX Ceramic Primer (3M Oral Care)	60	Variolink II, shade A3 (Ivoclar)	Shear bond strength (MPa)	24 h
Bottino [7]	2015	Brazil	8	Leucite feldspar-reinforced ceramic (VITA PM9 Vita Zahnfabrik; Bad Säckingen, Germany)	4, 5, and 9	Porcelain Primer (Bisco; Schaumburg, IL, USA)	60	Panavia F2.0 (Kuraray Noritake; Tokyo, Japan)	Microtensile bond strength (μTBS)	150 d followed by 12,000 thermocycles.
Kalavacharla [23]	2015	USA	10	Lithium-disilicate glass-ceramic (IPS e.max CAD, Ivoclar)	5 and 9.5	RelyX Ceramic Primer (3M Oral Care)	20 and 60	Z100, Shade A2 (3M Oral Care)	Shear bond strength (MPa)	10,000 thermocycles
Sundfeld [56]	2016	Brazil	13	Lithium-disilicate glass-ceramic (IPS e.max Press, Ivoclar)	5 and 10	Monobond-S (Ivoclar)	20	Variolink II, shade A2 (Ivoclar)	Microshear bond strength (mSBS)	24 h
Puppin-Rontani [44]	2017	Brazil	10	Lithium-disilicate glass-ceramic (IPS e.max Press, Ivoclar)	1, 2.5, 5, 7.5, and 10	RelyX Ceramic Primer (3M Oral Care)	20, 40, 60, 120, and 20 + 20	Variolink II, Shade Transparent (Ivoclar)	Microshear bond strength (µSBS)	24 h
Mokhtarpour [33]	2017	Iran	5	Lithium-disilicate glass-ceramic (IPS e.max CAD, Ivoclar) and feldspathic ceramic (VITA Mark II, Vita Zahnfabrik)	5 and 10	Clearfil Porcelain Bond Activator (Kuraray Noritake; Tokyo, Japan)	20, 60 and 120	Panavia F2.0 (Kuraray Noritake)	Microshear bond strength (µSBS)	Not reported
Sundfeld [57]	2018	Brazil	10	Lithium-disilicate (IPS e.max Press, Ivoclar)	1, 5, and 10	RelyX Ceramic Primer (3M Oral Care)	20	Experimental Resin composite bis-GMA/TEG-DMA and UDMA	Microtensile bond strength (μTBS)	24 h and 6 months
Prochnow [43]	2018	Brazil	10	Lithuim-disilicate IPS e.max CAD (Ivoclar)	1, 3, 5, and 10	Monobond-S (Ivoclar)	20	Dual-cure resin cement Multilink (Ivoclar)	Microshear bond strength (µSBS)	24 h, 150 days followed by 12,000 thermocycles
Colombo [9]	2019	Brazil	10	Polymer-infiltrated ceramic Enamic (Vita Zahnfabrik; Bad Säckingen, Germany); Composite Lava Ultimate (3M Oral Care; St Paul, MN, USA); Leucite-based glass-ceramic (IPS Empress CAD, Ivoclar); Lithium-disilicate IPS e.max CAD (Ivoclar)	5 and 10	Monobond N	20 and 60	Variolink N (Ivoclar)	Microshear bond strength (µSBS)	24 h
Straface [54]	2019	Switzer-land	10	Feldspathic ceramic (Vitablocs Mark II, Vita Zahnfabrik); Polymer-infiltrated ceramic Vita Enamic (Vita Zahnfabrik); Lithium-disilicate glass-ceramic (IPS e.max CAD, Ivoclar); Zirconia reinforced lithium-silicate ceramic (Vita Suprinity, Vita Zahnfabrik)	5 and 10	RelyX Ceramic Primer (3M Oral Care) Clearfil Ceramic Primer (Kuraray Noritake) Vita Adiva Cprime (VITA Zahnfabrik)	0, 5, 15, 30, and 60	RelyX Unicem 2 Automix (3M Oral Care) VITA Adiva S-Cem (VITA Zahnfabrik) Panavia V5 (Kuraray Noritake) VITA Adiva F-Cem (Vita Zahnfabrik)	Microshear bond strength (µSBS)	24 h
Lopes [29]	2019	Brazil	5	Lithium-disilicate glass-ceramic (IPS e.max CAD, (Ivoclar)	5, 9.5, 9.6, and 10	Monobond Plus (Ivoclar)	20	Variolink Veneer (Ivoclar)	Microshear bond strength (µSBS)	24 h
Veríssimo [63]	2019	Brazil	10	Lithium-disilicate glass-ceramic IPS e.max CAD (Ivoclar) Lithium-disilicate glass-ceramic IPS Empress CAD (Ivoclar) and Lithium-disilicate glass-ceramic IPS e.max Press (Ivoclar)	5 and 10	Prosil (FGM; Joinville, Brazil)	20 and 60	AllCem Dual resin cement (FGM)	Microshear bond strength (µSBS)	24 h followed by 10,000 thermocycles
Fonzar [20]	2020	Italy	15	Zirconia-reinforced lithium and lithium-disilicate glass-ceramic (IPS e.max CAD, Ivoclar)	4.9 and 9.5	Ultradent Silane (Ultradent)	20, 40, and 120	RelyX Unicem (3M Oral Care)	Microshear bond strength (µSBS)	24 h
Moura [32]	2020	Brazil	10	Feldspathic ceramic Vita Mark II, (Vita Zahnfabrik)	5 and 10	Prosil (FGM)	20 and 60	AllCem Dual resin cement (FGM)	Shear bond strength (MPa)	90 days
Azevedo [4]	2021	Brazil	10	Zirconia reinforced lithium-disilicate glass-ceramic (Vita Suprinity, Vita Zahnfabrik) and VITAblocs TriLuxe (Vita Zahnfabrik)	5 and 10	RelyX Ceramic Primer	20, 40, and 60	RelyX veneer cement (3M Oral Care)	Shear bond strength (MPa)	24 h and 16 months

### Selection Process

After searching, the articles were imported into Mendeley software^48^ (London, UK) for quality control. Duplicates were removed, titles and abstracts were checked in detail, and entries were categorized following the defined selection criteria. Articles were screened by three authors and discussed with another author in cases of disagreement. Eligible articles were selected for full-text reading and data extraction.

### Data Collection Process

Critical methodological data from included studies were extracted using a standardized form in Microsoft Office Excel 2013 software (Microsoft; Redmond, WA, USA). All trial documents contained: author names, publication year, country, number of samples, type of ceramic and brand, surface treatment protocol (HF etching and silane primer application), type of composite cement, test methods, and aging methods. In cases of missing information, the authors of the original papers were e-mailed twice, and the incomplete data were excluded if authors did not respond within one month.

### Risk of Bias Assessment

The risk of bias was evaluated by two authors according to the methods of other systematic reviews of in-vitro studies regarding bond strength of resin-based cements to glass-ceramic^11,27,52^ and using the Cochrane Collaboration’s tool.^22^ Using this instrument, parameters such as sample size calculation, comparable groups, detailed information regarding measurements, proper statistical analysis, adherence to manufacturer’s instructions, and single and/or blinded operator were evaluated. The risk of bias was classified as low, high, or unclear, and additional reviewers were consulted in cases of disagreement. A parameter was classified as low risk when detailed information was available, high risk if the information was not provided, and unclear risk when information was provided but not in detail. The corresponding authors were contacted if detailed information was lacking; the “unclear risk” classification was upheld if authors did not respond.

### Data Analysis

The meta-analyses were performed using Review Manager Software (version 5.4, Cochrane Collaboration; Oxford, UK). The global analysis was carried out using a random-effects model, and pooled-effect estimates were obtained by comparing the standardized mean difference between bond strengths derived for each HF concentration tested. p<0.05 was considered statistically significant.

## Results

### Study Selection

**Fig 1:**
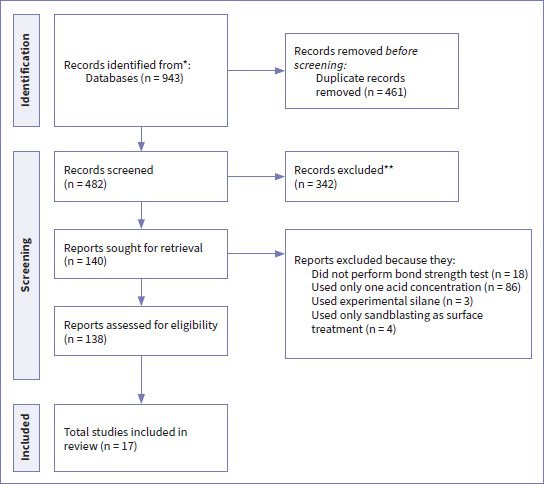
Search flowchart according to PRISMA guidelines.

Nine hundred forty-three (943) records were identified in our initial search: 304 from PubMed, 51 from Lilacs, 297 from Web of Science, and 291 from Scopus database (see Fig 1 for the PRISMA selection process). The duplicates were removed, and 342 records were excluded because they did not meet the eligibility criteria, leaving 138 articles for full-text reading. One hundred twenty-one (121) of these articles were subsequently removed. Based on the eligibility criteria, the remaining 17 studies were included in our final qualitative analysis, 12 of which were suitable for quantitative meta-analysis.

### Study Characteristics

The included studies were published from April 1989 to July 2021. Overall, the most frequently tested glass-ceramics were IPS e.Max CAD (Ivoclar; Schaan, Liechtenstein) (n = 8), IPS e.Max Press (Ivoclar) (n = 5), and VITA Mark II (Vita Zahnfabrik; Bad Säckingen, Germany) (n = 4) (Table 1). Regarding surface-etching protocols, HF ranged from 1% to 15% for 5 to 120 s of etching time, depending on the ceramic material. The most common glass–ceramic-specific silane primers were RelyX Ceramic Primer (3M Oral Care; St Paul, MN, USA) (n = 5), Monobond-S (Ivoclar Vivadent) (n = 2), Prosil (FGM; Joinville, Brazil) (n = 2), and Ultradent Silane (Ultradent; Jordan, UT, USA) (n = 2). The bond strength tests employed were microshear (n = 9), shear (n = 4), and microtensile (n = 4). Aging parameters (n = 15 total) ranged from 1 to 190 days and 10,000 to 12,000 thermocycles.

### Risk of Bias Assessment

**Fig 2:**
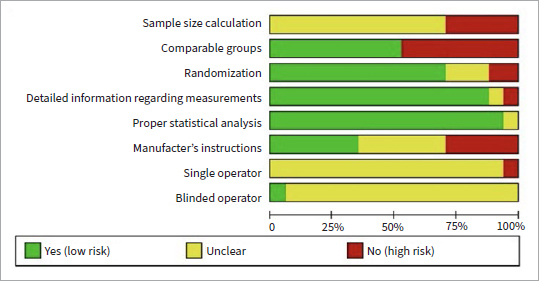
Distribution of risk of bias among the selected studies, according to pre-established criteria.

Figure 2 shows the articles classified according to the risk of bias. The highest risk (i.e., least reported) parameters were sample size calculation and single/blinded operator, while most studies showed a lower risk of bias as they met the criteria for the parameters comparable groups, randomization, detailed information regarding measurements, and proper statistical analysis.

### Synthesis of Results

**Fig 3:**
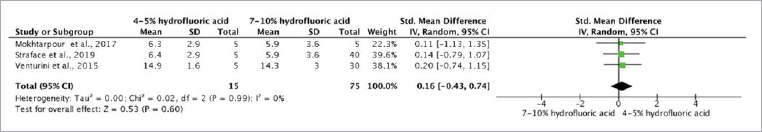
Forest plot comparing the bond strength to feldspathic ceramic when 4%-5% [HF] and 7%–10% [HF] was applied for 60 s, followed by glass-ceramic–specific silane primers.

**Fig 4:**
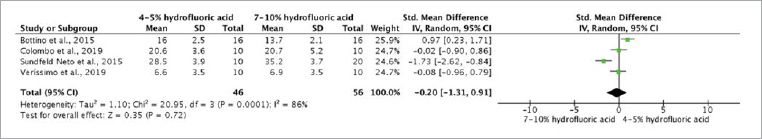
Forest plot comparing the bond strength to leucite ceramic when 4%–5% [HF] or 7%–10% [HF] was applied for 60 s, followed by glass-ceramic–specific silane primers.

**Fig 5:**
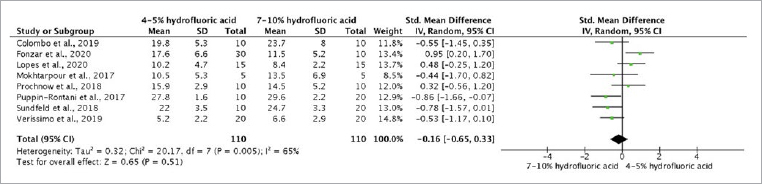
Forest plot comparing the bond strength to lithium-disilicate glass-ceramic when 4%–5% [HF] or 7%–10% [HF] was applied for 20 s, followed by glass-ceramic–specific silane primers.

**Fig 6:**
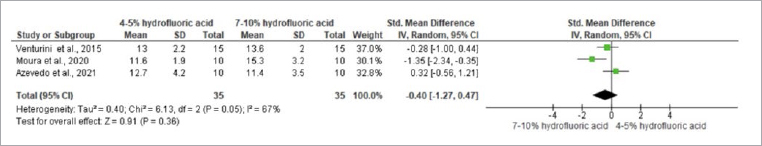
Forest plot comparing the bond strength to feldspathic ceramic when 4%–5% [HF] or 7%–10% [HF] was applied for 60 s, followed by glass-ceramic–specific silane primers after aging.

The meta-analysis was performed for only three types of glass-ceramics (feldspathic, leucite, and lithium-disilicate) because substantial methodological heterogeneity was observed for the other glass-ceramic types (resin-based hybrid materials and zirconia-reinforced lithium-silicate ceramics). The bond strength was not statistically different for a lower HF concentration (4% to 5%) as compared to 7% to 10% HF (Figs 3 to 6). A quantitative meta-analysis of the aging conditions was not performed due to methodological heterogeneity in the included studies.

In the qualitative analysis, it was observed that stable bonds can be formed with feldspathic ceramics after etching for 60 s with varying HF concentrations (3%, 5%, and 10%) and aging for 230 days and 12,000 thermocycles.^61^ Another study found that 10% HF produced higher shear bond strength than did 5% HF when etching for 60 or 120 s and aging for 90 days in distilled water.^33^ Azevedo et al^4^ reported that bond strength after etching with 5% or 10% HF for 20, 40, or 60 s generally decreases after 16 months of aging in water, although this is not true for feldspathic ceramics etched with 5% HF for 20 s. For leucite-based ceramics, Bottino et al^7^ reported that etching with different HF concentrations (4%, 5%, and 9%) for 60 s produced superior bond strength compared with the unetched control. However, in contrast to the control, the bond strength of the etched groups decreased significantly after aging in distilled water for 150 days followed by 12,000 thermal cycles.^7^

For lithium–disilicate-based ceramics, Kalavacharla et al^24^ reported that etching with 9.5% HF for 60 s produced higher bond strength after aging than did etching with 5% HF. Prochnow et al^44^ showed that 20 s of etching with 3%, 5%, and 10% HF did not affect the fatigue behavior of machined lithium-disilicate glass-ceramic crowns in cyclic load-to-failure tests (500,000 load pulses at a frequency of 20 Hz). However, the use of 3% HF for 20 s should be considered with caution, because it promotes slight topographical changes on the ceramic surface.^44^ Veríssimo et al^63^ reported higher bond strength for CAD/CAM ceramics after 20 s of etching with 5% HF compared to 20 s of etching with 10% HF. However, for pressed lithium-disilicate glass-ceramics, etching with 10% HF for 60 s produced higher bond strength after 10,000 thermocycles compared to CAD/CAM lithium-disilicate glass-ceramics.^63^

Our meta-analysis did not include resin-based hybrid materials or zirconia-reinforced lithium-silicate ceramics, but etching studies on polymer-infiltrated ceramics (Vita Enamic, Vita Zahnfabrik) revealed no statistically significant difference in bond strength when varying either HF (5%, 9%, and 10%) or etching time (15, 20, and 60 s).^9,54^ For zirconia-reinforced lithium-silicate ceramic (Vita Suprinity), Straface et al^54^ reported no significant difference in bond strength with HF etching at varying concentrations (5% and 9%) and etching times (15, 20, 30, and 60 s). In contrast, Fonzar et al^20^ demonstrated higher bond strengths when etching for 20 s with 4.9% HF compared to 9.5% HF. Furthermore, etching for >20 s did not increase bond strength.^20^

## Discussion

Surface treatment with hydrofluoric acid is used on glass-ceramics to promote strong, stable adhesive bonding in indirect restorations.^7,9,20,24,30,34,44,50,54,56,58,61,63^ This systematic review and meta-analysis of in-vitro studies compared the efficacy of glass-ceramic surface treatment with lower-concentration HF (<5%) etchants to that of higher-concentration HF (>5%) etchants. There were no statistically significant differences between treatments with low (4%–5%) and high (7%–10%) HF concentrations in short-term evaluations.

Ceramic restoration success and long-term stability depend on the bond quality between the ceramic and the composite cement,^6,59^ and several methods have been developed to test the bond strength and durability of the composite cement-ceramic interface. One of the means of increasing this bond strength is the application of silane, which promotes the formation of a siloxane chemical bond (Si-O-Si) between the hydroxyl groups (OH) present on the glass-ceramic and the alkoxy groups of the silanol unit (Si-OH) formed after acid-catalyzed hydrolysis of the silane coupling agent.^65^

The test method should predict clinical outcomes while being procedurally simple and repeatable by different laboratories.^49^ The methods reviewed here are among the most commonly used: microshear, microtensile, and shear bond strength tests.^59^ Microshear bond strength is tested in a small area with multiple bonded specimens on the same ceramic sample. However, this test may not measure the real interfacial bond strength because non-homogeneous stress distribution at the composite cement/ceramic interface can produce cohesive failures within the ceramic rather than at the bonded interface.^9,42^

The present meta-analysis compared the standardized mean difference among bond strengths derived from different bond strength tests, resulting in high data heterogeneity, as reported by other studies.^39,67^ Several aging methods were analyzed: storage for 24 h (most common),^9,20,45,50,54,56^ 6 months,^57^ or 90 days;^33^ and thermocycling for 12,000^7,61^ or 10,000^24,63^ cycles. Unfortunately, methodological heterogeneity in storage and thermocycling conditions prohibited a meta-analysis, and further studies are needed regarding the impact of aging methods on bond strength to glass-ceramics. Thermocycling can produce stress and volumetric changes at the bonded interface, and chemical hydrolysis of the polymeric adhesive layer’s hydroxyl, carboxyl, and ester groups can compromise bond strength.^10,17,50,60^

The methodological data used were suitable for examining glass-ceramics with three different compositions: feldspathic,^34,54,61^ leucite-based,^7,9,58^ and lithium-disilicate.^9,20,30, 34,44,45,58,63^ However, further laboratory and clinical studies are needed to determine the most appropriate surface treatments and HF concentration for polymer-infiltrated ceramics and zirconia-reinforced lithium-silicate glass-ceramics.

Etching with hydrofluoric acid is recommended for luting glass-ceramics,^53^ and works by the dissolution of the glassy phase to alter the ceramic microstructure. This dissolution promotes topographical changes on the ceramic surface, creating higher roughness and a favorable microstructure, which increases surface and energy area for bonding.^2,46,58^ Previous studies have demonstrated that higher HF concentrations (>5%) can cause over-dissolution of the glass matrix, which compromises mechanical properties especially in thin restorations.^15,16,68^ However, further studies using long-term aging procedures should be conducted to evaluate the effect on bond strength.

The negative effects of high-HF etchants are material dependent. Ceramics with a glassier surface matrix may be more affected because they contain silica. Silica becomes silicon tetrafluoride that reacts with hydrofluoric acid to form hexafluorosilicate, which when protonated forms tetrafluorosilicate acid that is easily removed with water.^31,36,55^

Higher HF concentrations (>5%) do not improve composite cement/ceramic bond strength. Our meta-analysis showed no statistically significant difference in bond strengths using lower (≤5%) vs higher HF (>5%) concentrations in short-term evaluations. Regarding aged bond strengths, we observed that 12,000 thermocycles produced stable bonds with feldspathic ceramic after etching for the 60 s at lower HF concentrations (3% and 5%).^61^ Leucite and lithium-disilicate glass-ceramics yielded bond stability after water storage for 150 days, followed by 12,000 thermal cycles and 10,000 cycles (5–50 °C/15 s dwell time), regardless of the etching time and HF strength used.^7,24^ Because hydrofluoric acid is a strong acid, the mechanism of etching is not based on acid corrosion of the ceramic’s glassy matrix, and therefore even lower HF concentrations have produced strong and stable bonding with composite cement.^25,59^ The glassy matrix is dissolved non-uniformly both superficially and internally, creating a deep, irregular etching pattern.^35,43^ In more deeply etched glass-ceramics, high-viscosity composite cement may penetrate poorly into these porosities, resulting in weak, unstable bonding.^2,14,15,37^ Thus, lower HF concentrations (≤5%) that produce shallower etching may produce effective resin bonding to glass-ceramics.

The meta-analysis should be interpreted with caution because it includes only short-term results from in-vitro studies (in-vivo studies are lacking). Therefore, further in-vitro studies with aged samples should be carried out, as well as clinical trials, to confirm and validate the findings of this study. The analysis demonstrates a high risk of bias regarding the parameters sample size calculation, operator characteristics, and manufacturer’s instructions. Furthermore, the meta-analysis included only three types of glass-ceramic (feldspathic, leucite, and lithium-disilicate) and excluded polymer-infiltrated ceramics and zirconia-reinforced lithium-silicate glass-ceramics due to substantial methodological heterogeneity.

Based on our meta-analysis, the bond strength produced by the etching of glass-ceramics with lower HF concentrations (<5%) does not differ from that of higher HF concentrations (>5%) in short-term evaluations.

## Supplementary Material

Search strategy up to July 2021

**Table:** 

Database	Search Term Key
Pubmed	((((((((((((“glass ceramic”[All Fields]) OR (“glass ceramics”[All Fields])) OR (glass ceramic[MeSH Terms])) OR (“lithia disilicate”[All Fields])) OR (lithia disilicate[MeSH Terms])) OR (lithium disilicate[MeSH Terms])) OR (“feldspathic”[All Fields])) OR (“feldspathic porcelain”[All Fields])) OR (“feldspathic ceramic”[All Fields])) OR (“leucite”[All Fields])) OR (leucite[MeSH Terms])) AND (((((((((“acid hydrofluoric”[All Fields]) OR (acid hydrofluoric[MeSH Terms])) OR (Acid, Hydrofluoric[MeSH Terms])) OR (“acid etching dental”[All Fields])) OR (“acid etching, dental”[MeSH Terms])) OR (Dental Acid Etching[MeSH Terms])) OR (Etching, Dental Acid[MeSH Terms])) OR (“surface treatment”[All Fields])) OR (“surface treatments”[All Fields]))) AND (((((((“bond strength”[All Fields]) OR (“shear bond strength”[All Fields])) OR (“microshear bond strength”[All Fields])) OR (“micro shear bond strength”[All Fields])) OR (“tensile bond strength”[All Fields])) OR (“microtensile bond strength”[All Fields])) OR (“micro tensile bond strength”[All Fields]))
Web of Science	TS = (“glass ceramic” OR “glass ceramics” OR “lithia disilicate” OR “lithium disilicate” OR “feldspathic” OR “feldspathic porcelain” OR “feldspathic ceramic” OR “leucite”) AND TS = (“acid hydrofluoric” OR “acid etching dental” OR “dental acid etching” OR “etching, dental acid” OR “surface treatment” OR “surface treatments”) AND TS = (“bond strength” OR “shear bond strength” OR “microshear bond strength” OR “micro shear bond strength” OR “tensile bond strength” OR “microtensile bond strength” OR “micro tensile bond strength”)
Scopus	TITLE-ABS-KEY(“glass ceramic” OR “glass ceramics” OR “lithia disilicate” OR “lithium disilicate” OR feldspathic OR “feldspathic porcelain” OR “feldspathic ceramic” OR leucite) AND TITLE-ABS-KEY(“acid hydrofluoric” OR “acid etching dental” OR “dental acid etching” OR “etching, dental acid” OR “surface treatment” OR “surface treatments”) AND TITLE-ABS-KEY(“bond strength” OR “shear bond strength” OR “microshear bond strength” OR “micro shear bond strength” OR “tensile bond strength” OR “microtensile bond strength” OR “micro tensile bond strength”)
LiLacs	(tw:(“glass ceramic” OR “glass ceramics” OR “lithia disilicate” OR “lithium disilicate” OR “feldspathic” OR “feldspathic porcelain” OR “feldspathic ceramic” OR “leucite”)) AND (tw:(“acid hydrofluoric” OR “acid etching dental” OR “dental acid etching” OR “etching, dental acid” OR “surface treatment” OR “surface treatments”)) AND (tw:(“bond strength” OR “shear bond strength” OR “microshear bond strength” OR “micro shear bond strength” OR “tensile bond strength” OR “microtensile bond strength” OR “micro tensile bond strength”))

## Clinical relevance:

In short-term evaluations, using a lower concentration of HF (≤5%) seems to be an effective etching treatment for bonding to glass-ceramic restorations.

## References

[ref1] Addison O, Marquis PM, Fleming GJP (2007). The impact of modifying alumina air abrasion parameters on the fracture strength of a porcelain laminate restorative material. Dent Mater.

[ref2] Addison O, Marquis PM, Fleming GJP (2007). The impact of hydrofluoric acid surface treatments on the performance of a porcelain laminate restorative material. Dent Mater.

[ref3] Albakry M, Guazzato M, Swain MV (2003). Biaxial flexural strength, elastic moduli, and x-ray diffraction characterization of three pressable all-ceramic materials. J Prosthet Dent.

[ref4] Azevedo VLB, de Castro EF, Bonvent JJ, de Andrade OS, Nascimento FD, Giannini M, Cavalli V (2021). Surface treatments on CAD/CAM glass-ceramics: Influence on roughness, topography, and bond strength. J Esthet Restor Dent.

[ref5] Belli R, Geinzer E, Muschweck A, Petschelt A, Lohbauer U (2014). Mechanical fatigue degradation of ceramics versus resin composites for dental restorations. Dent Mater.

[ref6] Bona AD, Shen CY, Anusavice KJ, Della Bona A, Shen CY, Anusavice KJ (2004). Work of adhesion of resin on treated lithia disilicate-based ceramic. Dent Mater.

[ref7] Bottino MAC, Snellaert A, Bergoli CD, Özcan M, Bottino MAC, Valandro LF (2015). Effect of ceramic etching protocols on resin bond strength to a feldspar ceramic. Oper Dent.

[ref8] Braga RR, Meira JBC, Boaro LCC, Xavier TA (2010). Adhesion to tooth structure: A critical review of “macro” test methods. Dent Mater.

[ref9] Colombo L do A, Murillo-Gómez F, De Goes MF (2019). Bond strength of CAD/CAM restorative materials treated with different surface etching protocols. J Adhes Dent.

[ref10] Crim GA, Swartz ML, Phillips RW (1985). Comparison of four thermocycling techniques. J Prosthet Dent.

[ref11] Da Rosa WLDO, Piva E, Da Silva AF (2015). Bond strength of universal adhesives: A systematic review and meta-analysis. J Dent.

[ref12] Darvell BW (2009). Adhesion strength testing –Time to fail or a waste of time?. J Adhes Sci Technol.

[ref13] Della Bona A, Anusavice KJ (2002). Microstructure, composition, and etching topography of dental ceramics. Int J Prosthodont.

[ref14] Della Bona A, Van Noort R (1995). Shear vs. tensile bond strength of resin composite bonded to ceramic. J Dent Res.

[ref15] Della Bona A, Anusavice KJ, Hood JAA (2002). Effect of ceramic surface treatment on tensile bond strength to a resin cement. Int J Prosthodont.

[ref16] De Melo RM, Valandro LF, Bottino MA, Melo RM de, Valandro LF, Bottino MA (2007). Microtensile bond strength of a repair composite to leucite-reinforced feldspathic ceramic. Braz Dent J.

[ref17] De Munck J, Van Landuyt K, Peumans M, Poitevin A, Lambrechts P, Braem M, Van Meerbeek B (2005). A critical review of the durability of adhesion to tooth tissue: Methods and results. J Dent Res.

[ref18] Denissen HW, El-Zohairy AA, van Waas MA, Feilzer AJ (2002). Porcelain-veneered computer-generated partial crowns. Quintessence Int.

[ref19] Denry I, Kelly JR (2014). Emerging ceramic-based materials for dentistry. J Dent Res.

[ref20] Fonzar RF, Goracci C, Carrabba M, Louca C, Ferrari M, Vichi A (2020). Influence of cid concentration and etching time on composite cement adhesion to lithium-silicate glass ceramics. J Adhes Dent.

[ref21] Gracis S, Thompson VP, Ferencz JL, Silva NR, Bonfante EA (2015). A new classification system for all-ceramic and ceramic-like restorative materials. Int J Prosthodont.

[ref22] Guess PC, Strub JR, Steinhart N, Wolkewitz M, Stappert CFJ (2009). All-ceramic partial coverage restorations-Midterm results of a 5-year prospective clinical splitmouth study. J Dent.

[ref23] Higgins JPT, Altman DG, Gøtzsche PC, Jüni P, Moher D, Oxman AD, Savovic J, Schulz KF, Weeks L, Sterne JA (2011). The Cochrane Collaboration’s tool for assessing risk of bias in randomised trials. BMJ.

[ref24] Kalavacharla VK, Lawson NC, Ramp LC, Burgess JO (2015). Influence of etching protocol and silane treatment with a universal adhesive on lithium disilicate bond strength. Oper Dent.

[ref25] Kelly JR, Benetti P (2011). Ceramic materials in dentistry: Historical evolution and current practice. Aust Dent J.

[ref26] Kollmuss M, Kist S, Goeke JE, Hickel R, Huth KC (2016). Comparison of chairside and laboratory CAD/CAM to conventional produced all-ceramic crowns regarding morphology, occlusion, and aesthetics. Clin Oral Investig.

[ref27] Lima RBW, Barreto SC, Hajhamid B, de Souza GM, de Goes MF (2019). Effect of cleaning protocol on silica deposition and silica-mediated bonding to Y-TZP. Dent Mater.

[ref28] Lima RBW, Troconis CCM, Moreno MBP, Murillo-Gómez F, De Goes MF (2018). Depth of cure of bulk fill resin composites: A systematic review. J Esthet Restor Dent.

[ref29] Lin W-S, Ercoli C, Feng C, Morton D (2012). The effect of core material, veneering porcelain, and fabrication technique on the biaxial flexural strength and Weibull analysis of selected dental ceramics. J Prosthodont Esthet Reconstr Dent.

[ref30] Lopes GC, Perdigão J, Baptista D, Ballarin A (2019). Does a self-etching ceramic primer improve bonding to lithium disilicate ceramics? Bond strengths and FESEM analyses. Oper Dent.

[ref31] Matinlinna JP, Vallittu PK (2007). Bonding of resin composites to etchable ceramic surfaces –An insight review of the chemical aspects on surface conditioning. J Oral Rehabil.

[ref32] Meyer Filho A, Vieira LCC, Araújo É, Monteiro S (2004). Effect of different ceramic surface treatments on resin microtensile bond strength. J Prosthodont.

[ref33] Moura DMD, Araújo AMM de, Souza KB de, Veríssimo AH, Tribst JPM, Souza ROAE (2020). Hydrofluoric acid concentration, time, and use of phosphoric acid on the bond strength of feldspathic ceramics. Braz Oral Res.

[ref34] Mokhtarpour F, Alaghehmand H, Khafri S (2017). Effect of hydrofluoric acid surface treatments on micro-shear bond strength of CAD/CAM ceramics. Electron Physician.

[ref35] Murillo-Gomez F, Palma-Dibb RG, De Goes MF (2018). Effect of acid etching on tridimensional microstructure of etchable CAD/CAM materials. Dent Mater.

[ref36] Murillo-Gómez F, De Goes MF (2019). Bonding effectiveness of tooth-colored materials to resin cement provided by self-etching silane primer after short- and long-term storage. J Prosthet Dent.

[ref37] Naves LZ, Soares CJ, Moraes RR, Gonçalves LS, Sinhoreti MAC, Correr-Sobrinho L (2010). Surface/interface morphology and bond strength to glass ceramic etched for different periods. Oper Dent.

[ref38] Nejatidanesh F, Amjadi M, Akouchekian M, Savabi O (2015). Clinical performance of CEREC AC Bluecam conservative ceramic restorations after five years –A retrospective study. J Dent.

[ref39] Nogueira I de O, Oliveira PFG de, Magno MB, Ferreira DMTP, Maia LC, Rabello TB (2021). Does the application of an adhesive layer improve the bond strength of etched and silanized glass-ceramics to resin-based materials? A systematic review and meta-analysis. J Prosthet Dent.

[ref40] Özcan M, Vallittu PK (2003). Effect of surface conditioning methods on the bond strength of luting cement to ceramics. Dent Mater.

[ref41] Page MJ, McKenzie JE, Bossuyt PM, Boutron I, Hoffmann TC, Mulrow CD, Shamseer L, Tetzlaff JM, Akl EA, Brennan SE, Chou R, Glanville J, Grimshaw JM, Hróbjartsson A, Lalu MM, Li T, Loder EW, Mayo-Wilson E, McDonald S, McGuinness LA, Stewart LA, Thomas J, Tricco AC, Welch VA, Whiting P, Moher D (2021). The PRISMA 2020 statement: An updated guideline for reporting systematic reviews. BMJ.

[ref42] Placido E, Meira JBC, Lima RG, Muench A, Souza RM, Ballester RY (2007). Shear versus micro-shear bond strength test: A finite element stress analysis. Dent Mater.

[ref43] Posritong S, Souto Borges AL, Chu T-MG, Eckert GJ, Bottino MA, Bottino MC (2013). The impact of hydrofluoric acid etching followed by unfilled resin on the biaxial strength of a glass-ceramic. Dent Mater.

[ref44] Prochnow C, Venturini AB, Guilardi LF, Rocha Pereira GK, Lima Burgo TA, Bottino MC, Kleverlaan CJ, Valandro LF (2018). Hydrofluoric acid concentrations: Effect on the cyclic load-to-failure of machined lithium disilicate restorations. Dent Mater.

[ref45] Puppin-Rontani J, Sundfeld D, Costa AR, Correr AB, Puppin-Rontani RM, Borges GA, Sinhoreti MAC, Correr-Sobrinho L (2017). Effect of hydrofluoric acid concentration and etching time on bond strength to lithium disilicate glass ceramic. Oper Dent.

[ref46] Ramakrishnaiah R, Alkheraif AA, Divakar DD, Matinlinna JP, Vallittu PK (2016). The effect of hydrofluoric acid etching duration on the surface micromorphology, roughness, and wettability of dental ceramics. Int J Mol Sci.

[ref47] Ramos NDC, Campos TMB, Paz ISD La, MacHado JPB, Bottino MA, Cesar PF, Melo RM (2016). Microstructure characterization and SCG of newly engineered dental ceramics. Dent Mater.

[ref48] Reis MAF, Favretto J, Favretto NM, Favretto LMH, Santos RP (2022). Knowledge management in the classroom using Mendeley technology. J Acad Librarian.

[ref49] Roeder L, Pereira PNR, Yamamoto T, Ilie N, Armstrong S, Ferracane J (2011). Spotlight on bond strength testing –Unraveling the complexities. Dent Mater.

[ref50] Sai K, Shimamura Y, Takamizawa T, Tsujimoto A, Imai A, Endo H, Barkmeier WW, Latta MA, Miyazaki M (2016). Influence of degradation conditions on dentin bonding durability of three universal adhesives. J Dent.

[ref51] Saracoglu A, Cura C, Cotert HS (2004). Effect of various surface treatment methods on the bond strength of the heat-pressed ceramic samples. J Oral Rehabil.

[ref52] Soares CJ, Soares PV, Pereira JC, Fonseca RB (2005). Process of ceramic and laboratory-processed composite restorations : a literature review. J Esthet Restor Dent.

[ref53] Soares FZM, Follak A, da Rosa LS, Montagner AF, Lenzi TL, Rocha RO (2016). Bovine tooth is a substitute for human tooth on bond strength studies: A systematic review and meta-analysis of in vitro studies. Dent Mater.

[ref54] Straface A, Rupp L, Gintaute A, Fischer J, Zitzmann NU, Rohr N (2019). HF etching of CAD/CAM materials: influence of HF concentration and etching time on shear bond strength. Head Face Med.

[ref55] Sudré JP, Salvio LA, Baroudi K, Sotto-Maior BS, Melo-Silva CLCL, Souza Picorelli Assis NM (2020). Influence of surface treatment of lithium disilicate on roughness and bond strength. Int J Prosthodont.

[ref56] Sundfeld D, Correr-Sobrinho L, Pini NIP, Costa AR, Sundfeld RH, Pfeifer CS, Martins LR (2016). The effect of hydrofluoric acid concentration and heat on the bonding to lithium disilicate glass ceramic. Braz Dent J.

[ref57] Sundfeld D, Palialol ARM, Fugolin APP, Ambrosano GMB, Correr-Sobrinho L, Martins LRM, Pfeifer C (2018). The effect of hydrofluoric acid and resin cement formulation on the bond strength to lithium disilicate ceramic. Braz Oral Res.

[ref58] Sundfeld Neto D, Naves LZ, Costa AR, Correr AB, Consani S, Borges GA, Correr-Sobrinho L (2015). The effect of hydrofluoric acid concentration on the bond strength and morphology of the surface and interface of glass ceramics to a resin cement. Oper Dent.

[ref59] Tian T, Tsoi JKH, Matinlinna JP, Burrow MF (2014). Aspects of bonding between resin luting cements and glass ceramic materials. Dent Mater.

[ref60] Van Landuyt KL, Snauwaert J, De Munck J, Peumans M, Yoshida Y, Poitevin A, Coutinho E, Suzuki K, Lambrechts P, Van Meerbeck B (2007). Systematic review of the chemical composition of contemporary dental adhesives. Biomaterials.

[ref61] Venturini AB, Prochnow C, Rambo D, Gundel A, Valandro LF (2015). Effect of hydrofluoric acid concentration on resin adhesion to a feldspathic ceramic. J Adhes Dent.

[ref62] Venturini AB, Prochnow C, May LG, Bottino MC, Valandro LF (2015). Influence of hydrofluoric acid concentration on the flexural strength of a feldspathic ceramic. J Mech Behav Biomed Mater.

[ref63] Veríssimo AH, Moura DMD, Tribst JPM, Araújo AMM de, Leite FPP, Souza ROAE (2019). Effect of hydrofluoric acid concentration and etching time on resin-bond strength to different glass ceramics. Braz Oral Res.

[ref64] Veríssimo AH, Duarte Moura DM, de Oliveira Dal Piva AM, Bottino MA, de Fátima Dantas de Almeida L, da Fonte Porto Carreiro A, Assunção E, Souza RO (2020). Effect of different repair methods on the bond strength of resin composite to CAD/CAM materials and microorganisms adhesion: An in situ study. J Dent.

[ref65] Warring SL, Beattie DA, McQuillan AJ (2016). Surficial siloxane-to-silanol interconversion during room-temperature hydration/dehydration of amorphous silica films observed by ATR-IR and TIR-Raman. Langmuir.

[ref66] Walker E, Hernandez AV, Kattan MW (2008). Meta-analysis: Its strengths and limitations. Cleve Clin J Med.

[ref67] Zhang Y, Kelly JR (2017). Dental ceramics for restoration and metal veneering. Dent Clin North Am.

[ref68] Zogheib LV, Della Bona A, Kimpara ET, McCabe JF (2011). Effect of hydrofluoric acid etching duration on the roughness and flexural strength of a lithium disilicate-based glass ceramic. Braz Dent J.

